# Selective ATP-Binding Cassette Subfamily C Gene Expression and Proinflammatory Mediators Released by BEAS-2B after PM_2.5_, Budesonide, and Cotreated Exposures

**DOI:** 10.1155/2017/6827194

**Published:** 2017-08-16

**Authors:** Jarline Encarnación-Medina, Rosa I. Rodríguez-Cotto, Joseph Bloom-Oquendo, Mario G. Ortiz-Martínez, Jorge Duconge, Braulio Jiménez-Vélez

**Affiliations:** ^1^School of Pharmacy, University of Puerto Rico, Medical Science Campus, San Juan, PR, USA; ^2^Center for Environmental and Toxicological Research, San Juan, PR, USA; ^3^School of Medicine, University of Puerto Rico, San Juan, PR, USA; ^4^Institute of Biomedical and Forensic Sciences Research of Puerto Rico Inc. (IBFSR), San Juan, PR, USA

## Abstract

ATP-binding cassette subfamily C (ABCC) genes code for phase III metabolism proteins that translocate xenobiotic (e.g., particulate matter 2.5 (PM_2.5_)) and drug metabolites outside the cells. IL-6 secretion is related with the activation of the ABCC transporters. This study assesses ABCC1–4 gene expression changes and proinflammatory cytokine (IL-6, IL-8) release in human bronchial epithelial cells (BEAS-2B) exposed to PM_2.5_ organic extract, budesonide (BUD, used to control inflammation in asthmatic patients), and a cotreatment (Co-T: PM_2.5_ and BUD). A real-time PCR assay shows that ABCC1 was upregulated in BEAS-2B exposed after 6 and 7 hr to PM_2.5_ extract or BUD but downregulated after 6 hr of the Co-T. ABCC3 was downregulated after 6 hr of BUD and upregulated after 6 hr of the Co-T exposures. ABCC4 was upregulated after 5 hr of PM_2.5_ extract, BUD, and the Co-T exposures. The cytokine assay revealed an increase in IL-6 release by BEAS-2B exposed after 5 hr to PM_2.5_ extract, BUD, and the Co-T. At 7 hr, the Co-T decreases IL-6 release and IL-8 at 6 hr. In conclusion, the cotreatment showed an opposite effect on exposed BEAS-2B as compared with BUD. The results suggest an interference of the BUD therapeutic potential by PM_2.5_.

## 1. Introduction

Airborne particulate matter 2.5 (PM_2.5_) is within the most regulated pollutant worldwide due to its intrinsic physicochemical properties that make it hazardous to the respiratory and cardiovascular system [1]. PM_2.5_ is composed of inorganic compounds (metals and minerals) and organic pollutants (bacterial endotoxins, fungal spores, pollen fragments, polycyclic aromatic hydrocarbons (PAH), and carbonaceous elements) [2–7]. Due to the small diameter of these particles (2.5 *μ*m), once they are inhaled through the respiratory system, they are easily transported into the arterial circulation [4, 8, 9]. The inhaled components can narrow the airways and induce inflammatory responses that can aggravate any existing respiratory conditions such as asthma or chronic obstructive pulmonary disease (COPD) [5, 10–14]. PM_2.5_ has been widely studied [4, 10, 15] and is extremely regulated in the United States by the Environmental Protection Agency (USEPA); however, the metabolism of this xenobiotic in lung cells [16–18] and its effect in the local immune response have not been fully elucidated.

Previous studies have shown that the antioxidant response to PM_2.5_ in bronchial epithelial cells (e.g., BEAS-2B) is activated through nuclear factor-erythroid 2-related factor 2 (Nrf2) which leads to the activation of mRNAs for heme oxygenase (HMOX1) and glutathione-S-transferase (GSTP1) that code for enzymes responsible for activating the second phase of the metabolism [4, 19, 20]. In addition to the cell detoxification mechanism, bronchial epithelial cells are responsible for modulating the local immune response against foreign agents like PM_2.5_ [21, 22]. As part of the local immune response of BEAS-2B against PM exposure, toll-like receptor- (TLR-) 2 and 4 activation followed by secretion of interleukins 6 (IL-6) and 8 (IL-8) has been reported [3, 13, 23, 24]. Nfr2 transcription factor activation as well as the IL-6 secretion are related with the activation of the ATP-binding cassette subfamily C (ABCC) transporters [25]. Since we have previously demonstrated that Nrf2 and IL-6 are induced in BEAS-2B upon PM_2.5_ exposure [4, 26, 27], we expect to find an upregulation of the ABCC1–4 genes upon the PM_2.5_ exposure. To the best of our knowledge, there is no information about the ABCC1–4 gene expression in BEAS-2B exposed to PM_2.5_, which involves the third phase of the xenobiotic metabolism.

The third phase of the cell metabolism is depending on the ATP-binding cassette subfamily C genes encoding the multidrug-resistant proteins (MRPs). The ABCC1–4 genes encode membrane proteins involved in various physiological events and function as efflux pumps of metabolic waste products (e.g., glutathione (GSH), glucuronide, and sulfate conjugates) [28–31]. Overexpression of these transporters can be responsible for drug inefficacy, which occurs when the cell pumps out the drug without any selectivity. Activation of ABCC1–4 has been associated with the presence of corticosteroids, and its derivate products have also been identified as potential regulators or precursors of ABCC1–4 genes [28, 32, 33]. Thus, we also expect regulation to be affected after budesonide (BUD) corticosteroid treatment in BEAS-2B. Moreover, a cotreatment (Co-T) of PM_2.5_ and corticosteroids was performed to study the efficiency of the corticosteroids upon PM_2.5_ exposure using the BEAS-2B.

Bronchial epithelial cells are one of the first targets for environmental and inhaled drug metabolism due to its location in the respiratory tract. These cells play an important role in the xenobiotic and drug fate that includes the clearance of these molecules through a set of enzymes (phase II) and ABCC1–4 gene activation. Our results will allow us to provide a background about the ABCC gene regulation that may happen once PM_2.5_, corticosteroids, or both interact in bronchial epithelial cells that might decide the fate of these molecules. There is very limited research on the effects of PM_2.5_ or BUD on the ABCC1–4 transporters and even less on the effects of the combined exposure of these treatments. Our experimental approach included the use of the most prescribed synthetic corticosteroid, BUD [34]. It is important to highlight that no therapeutic drug has been proven to be effective against air pollution-induced asthma or COPD. BUD is known to reduce ozone-induced inflammation, but does not protect against decreased lung function [35, 36]. However, the primary role of corticosteroids such as BUD consists in downregulating inflammatory cytokines to reduce the level of concomitant inflammation. As previously mentioned, it has been demonstrated that PM_2.5_ induces IL-6 and IL-8 in human bronchial epithelial cells [2–5, 10, 37]. Therefore, the aim of this study was to assess the response induced by PM_2.5_ and BUD in BEAS-2B by measuring the levels of IL-6 and IL-8 through a specific time course at 5, 6, and 7 hr. These data will allow us to indirectly determine the drug effect. Our results provide additional information on the cytotoxic effects of PM_2.5_ and BUD to BEAS-2B. In addition, we aimed to elucidate the effects of these treatments on the expression of ABCC1–4 genes at 5, 6, and 7 hours after exposure. Our results provide a guide towards the elaboration of a biochemical framework to explain the role of PM_2.5_, BUD, and the Co-T on MRP regulation.

## 2. Materials and Methods

### 2.1. Site and Sample Selection

PM_2.5_ samples were collected in circular Teflon filters by the Puerto Rico Environmental Quality Board, which maintains a net of monitoring stations at various strategic points into the island to monitor the air quality. The designated station is in Guayama, Puerto Rico. Data from Fajardo Puerto Rico, a reference location, was used for comparison of PM_2.5_ toxicity of the two sites.

### 2.2. Sampler Collection Equipment and PM_2.5_ Extractions

Teflon filters were set for 24 hr for the entire period of January 01, 2008 to December 31, 2008 in a PM_2.5_ sampler air collector (R&P model 2025), previously programmed with a standard method developed by the USEPA specifications. The filters were exchanged after the 24 hr completion. Once the collection was completed, the filters were labeled with a number referring to the collection day and place and identified as PM_2.5_. Teflon filters were stably weighed, following the USEPA methodology (Environmental Protection Agency, 1998). All glassware was washed using a modified cleanup procedure that includes the acidic treatment [38]. The collected PM_2.5_ filters were extracted during 15 minutes with 180 ml of hexane/acetone 1 : 1 (Fisher) using a microwave-assisted extraction system (MAE) (Ethos plus Microwave Labstation). Sample digestion was performed according to Alvarez-Avilés et al. [39]. Briefly, after the digestion with MAE, the large amount of solvent was removed with a gentle steam of nitrogen across the surface of the conic vials, using a tank of compressor gas. The protocol had the following constant parameters: 80°C and 1000 W and time points: T_1_ = 10 min and T_2_ = 5 min. The analytical weight of the samples was performed following gravimetric method. The organic extracts were resuspended in dimethyl sulfoxide (DMSO, Molecular Biology, Fisher) to a concentration of 100 mg/ml as a final stock solution. An organic extract composite corresponding to July 2008 filters was prepared and then stored at −20°C. The PM_2.5_ extract is the limited reagent of the study; therefore, the experiments were designed only to answering the proposed questions.

### 2.3. Cell Culture

Human bronchial epithelial cells (BEAS-2B, ATCC® #CRL-9609™) were cultured and maintained in keratinocyte growth medium 2 (KGM-2, Walkersville, MD). The cells were incubated in a humidified atmosphere of 5% CO_2_ at 37°C. Before each biological assay, the cells were seeded at a density of 5 × 10^4^ cells/well into 96-well plates and incubated for 24 hr.

### 2.4. Cell Viability Assay

The neutral red bioassay (Sigma, St. Louis, MO) was performed to obtain the lethal dose (LD_50_) for 50% mortality of the different agents. The cell treatments included PM_2.5_ (25, 50, 75, and 100 *μ*g/ml), GSH, Sigma, MO (5, 10, 25, 75, 100, 250, and 500 *μ*g/ml), and the suspension BUD (0.01, 0.03, 0.05, and 0.1 *μ*g/ml). The Co-T concentration was obtained from the nontoxic concentrations of PM_2.5_ (25 *μ*g/ml) and Bud (0.05 *μ*g/ml). After 24 hr, the supernatants with the treatments were removed. The neutral red dye was added for 3 hr. After removing the dye, the cells were fixed with a 0.5% formaldehyde/1% calcium chloride solution and rinsed with 1x phosphate-buffered saline (PBS) to eliminate unfixed cells, excess dye, and formaldehyde residues. The BEAS-2B were then lysed using a 1% acetic acid/50% ethanol solution. Cell viability was determined with spectrophotometry at 540 nm using an Ultramark microplate reader (Bio-Rad, Richmond, CA, USA). Triton-X treatment (25 *μ*g/ml) was used as a positive control. Values less than 80% cell viability were considered cytotoxic. The different solvents including media, DMSO, and H_2_O were used as negative controls.

### 2.5. Real-Time Polymerase Chain Reaction (PCR)

Gene expression assay validation was performed using TaqMan® (Applied Biosystems, CA). A calibration curve was constructed using the GSH exposure (50 *μ*g/ml); GSH is a positive inductor of the target gene, ATP-binding cassette C subfamily (ABCC1–4) [38–41]. Cells were exposed to PM_2.5_ extract (25 *μ*g/ml), BUD (0.05 *μ*g/ml), and the Co-T at different time points (5, 6, and 7 hr). Total RNA was extracted using TRIZOL reagent (Invitrogen, CA). The high-capacity complementary DNA (cDNA) reverse transcription kit (Applied Biosystems, CA) was used to synthesize cDNA. Quantitative florescent amplification of cDNA of ABCC1 (Hs01561502_m1), ABCC3 (Hs00978473_ml), and ABCC4 (Hs00988717_m1) was performed using TaqMan Gene Expression Assays (Applied Biosystems, CA). The real-time polymerase chain reaction (RTPCR) was conducted in a StepOne Real-Time PCR System (Applied Biosystems, CA). *β*-Actin (Hs03023943_ml) was used as a housekeeping gene to normalize the target genes.

### 2.6. Cytokine Assay

The cytokines were obtained from cell supernatant collected after 5, 6, and 7 hr of treatment with PM_2.5_ extract, BUD, and the Co-T and transferred to a 96-well plate. A simultaneous and quantitative detection of the proinflammatory mediators IL-6, IL-8, IL-10, and IL-13 was performed using a multiplex bead assay (Multianalyte Profiling Kit from R&D Systems, Minneapolis, MN) and a Luminex 100 (Luminex Corp., Austin, TX, USA) instrument according to the manufacturer's instructions. Lipopolysaccharide (LPS) at 10 *μ*g/ml was used as a positive control in the assay.

### 2.7. Statistical Analyses

To assess the differences between individual groups, the unpaired Student's *t*-test was employed. The criterion for statistical significance was set at ^∗∗∗^*p* < 0.001, ^∗∗^*p* < 0.01, and ^∗^*p* < 0.05. Statistical analyses were performed using the GraphPad InStat 3 software. Analyses were based on three independent experiments.

## 3. Results

### 3.1. Cell Viability Assay

**Figure 1:**
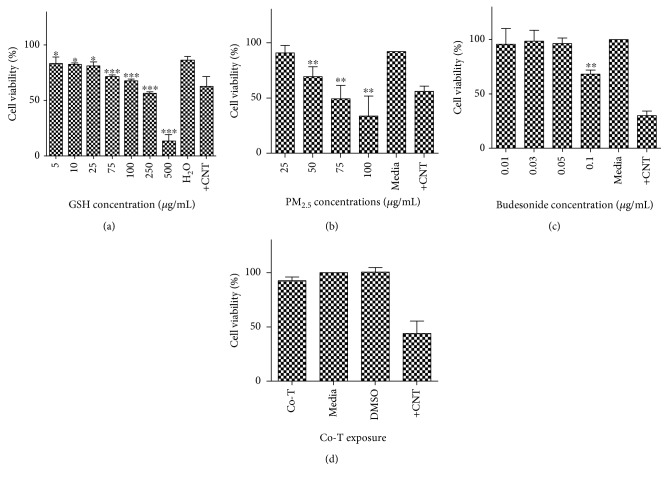
Cell viability assays for GSH, PM_2.5_ extracts, budesonide (BUD), and the Co-T. BEAS-2B were treated for 24 hr with each treatment. (a) The 25 *μ*g/ml dose of GSH was selected as the highest nontoxic concentration and used as the positive control for ABCC1–4 gene expression experiments. (b) The highest nontoxic concentration of PM_2.5_ extracts selected for further experiments was 25 *μ*g/ml. (c) The highest nontoxic concentration for the BUD exposure was 0.05 *μ*g/ml. (d) Using the nontoxic concentrations of PM_2.5_ and BUD, a Co-T was established. Bars represent the mean cell viability of three independent experiments (*N* = 3). Triton-X (25 *μ*g/ml) was used as positive control (+CNT). Asterisks denote statistical significance: ^∗∗∗^*p* < 0.001, ^∗∗^*p* < 0.01, ^∗^*p* < 0.05.

The toxicity of GSH, PM_2.5_ extracts, BUD, and the Co-T was evaluated in BEAS-2B. From these experiments, we selected the nontoxic concentrations to be used for gene expression assays and measurement of cytokine levels. A linear relationship between GSH concentration and cell viability was obtained with an estimated lethal dose (LD_50_) of 253 *μ*g/ml in BEAS-2B. The highest toxicity was observed at 250 and 500 *μ*g/ml GSH, reducing cell viability to 56.22% and 13.13%, respectively (*p* < 0.001) (Figure 1(a)). These results were used to establish a nontoxic concentration (25 *μ*g/ml) for the positive control of ABCC gene induction. Dose-response experiments for the PM_2.5_ extracts indicate that concentrations above 25 *μ*g/ml are significantly toxic to cells (Figure 1(b)). Our results show that 50, 75, and 100 *μ*g/ml PM_2.5_ extracts reduce cell viability to almost 69.36%, 49.32%, and 33.60%, respectively (*p* < 0.01). An inverse relationship between PM_2.5_ extract concentration and cell viability was observed. From these results, the LD_50_ for PM_2.5_ extract was calculated to be 76.7 *μ*g/ml. Concentrations of BUD above 0.1 *μ*g/ml caused toxic effects on BEAS-2B. (Figure 1(c)). A dose-response curve was obtained showing an inverse relationship between cell viability and BUD increasing concentration. Although higher concentrations were not included, an LD_50_ of 0.17 *μ*g/ml was extrapolated. The nontoxic concentrations of 25 *μ*g/ml for PM_2.5_ extract and 0.05 *μ*g/ml for BUD were selected for the subsequent experiments as well as to create the Co-T concentration. The cotreatment did not induce any cell toxicity at the concentration tested (Figure 1(d)).

### 3.2. Partial Time Course of ATP-Binding Cassette Genes (ABCC 1, 2, 3, and 4) Expression with Treatments

**Figure 2:**
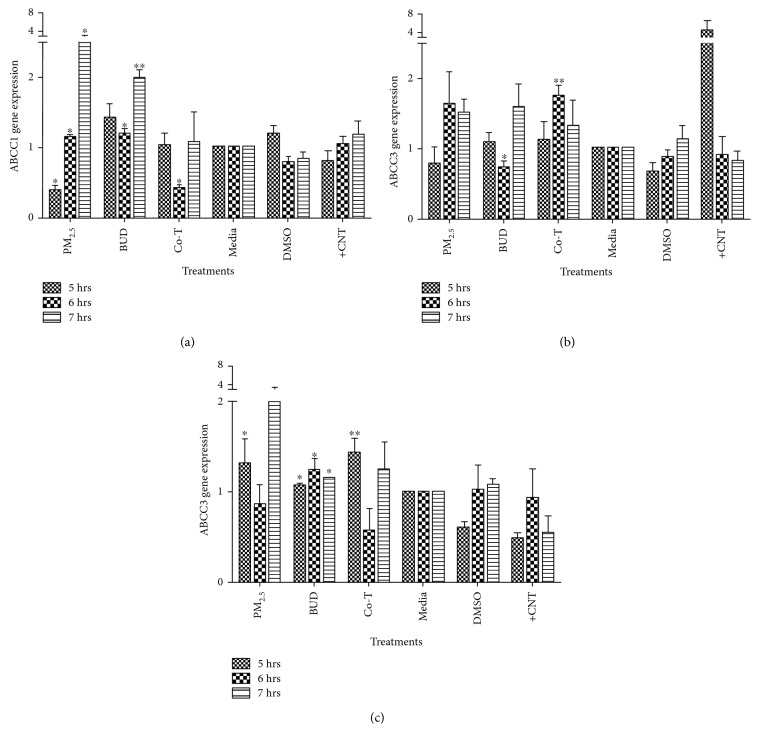
Time course of ABCC1, ABCC3, and ABCC4 mRNA in BEAS-2B with the different treatments: ABCC1–4 mRNA response to PM_2.5_ extract (25 *μ*g/ml), budesonide (BUD) (0.05 *μ*g/ml), and to the cotreatment at various time points (5, 6, and 7 hr) of exposure. Bars represent mean cell viability ± SEM of three independent experiments (*N* = 3); ^∗∗^*p* < 0.01, ^∗^*p* < 0.05. Asterisks over the bar indicate comparison of the treatments with the solvent (media or DMSO). GSH 25 *μ*g/ml was used as positive control.

A partial time course of the ABCC1–4 gene expression in BEAS-2B exposed to GSH, PM_2.5_ extract, BUD, and the Co-T was performed to determine any alterations on their regulation due to these exposures. The ABCC2 was not induced by any of these treatments. PM_2.5_ extract induced an upregulation of ABCC1 and ABCC4 gene expression at various time points in BEAS-2B (Figures 2(a) and 2(c)). Significant inductions of ABCC1 were found when comparing DMSO-treated cells with PM_2.5_ extract-treated cells after 6 and 7 hr of exposure (*p* < 0.05). There is a direct time response relationship of ABCC1 expression and PM_2.5_ extract exposure in BEAS-2B (Figure 2(a)). A suppression of the ABCC1 gene was observed after 5 hr of PM_2.5_ extract exposure when compared with the DMSO control (*p* < 0.05). The ABCC3 expression was also evaluated at 5, 6, and 7 hr after the PM_2.5_ extract exposure; however, no significant differences were detected when comparing with control cells (Figure 2(b)). As for the ABCC4, the peak induction of gene expression was found at 7 hr although no statistical significance was observed. The second highest expression was seen at 5 hr, which was statistically significant (Figure 2(c)).

Overall, BUD treatment induced the expression of ABCC1 and ABCC4 at the time points studied (Figures 1(a) and 1(c)). Specifically, ABCC1's highest induction was observed at 7 hr of exposure (*p* < 0.01) (Figure 2(a)). BUD suppressed the expression of ABCC3 at 6 hr (Figure 2(b)). ABCC3 expression was unaltered at 5 hr of exposure; however, its highest expression was seen at 7 hr of BUD treatment although it was not significantly different when compared with that of control cells (Figure 2(b)). The highest induction of ABCC4 was seen at 6 hr of BUD exposure (*p* < 0.05) (Figure 2(c)).

The Co-T did not significantly alter ABCC1 expression at 5 or 7 hr of exposure (Figure 2(a)). However, a significant reduction of ABCC1 expression was observed after 6 hr (Figure 2(a)). Both PM_2.5_ extract and BUD independently increased ABCC1 expression significantly at 6 hr, but the Co-T had an opposite effect. The gene expression of ABCC3 peaked at 6 hr (*p* < 0.01), but no differences were found at neither 5 nor 7 hr of the Co-T (Figure 2(b)). The ABCC4 showed a significant increase of expression after 5 hr of the Co-T (*p* < 0.01) (Figure 2(c)). This induction of ABCC4 at 5 hr is analogous to the results obtained by the PM_2.5_ extract and BUD exposure (Figure 2(c)).

### 3.3. Partial Time Course of Cytokine Measurements after Treatments

**Figure 3:**
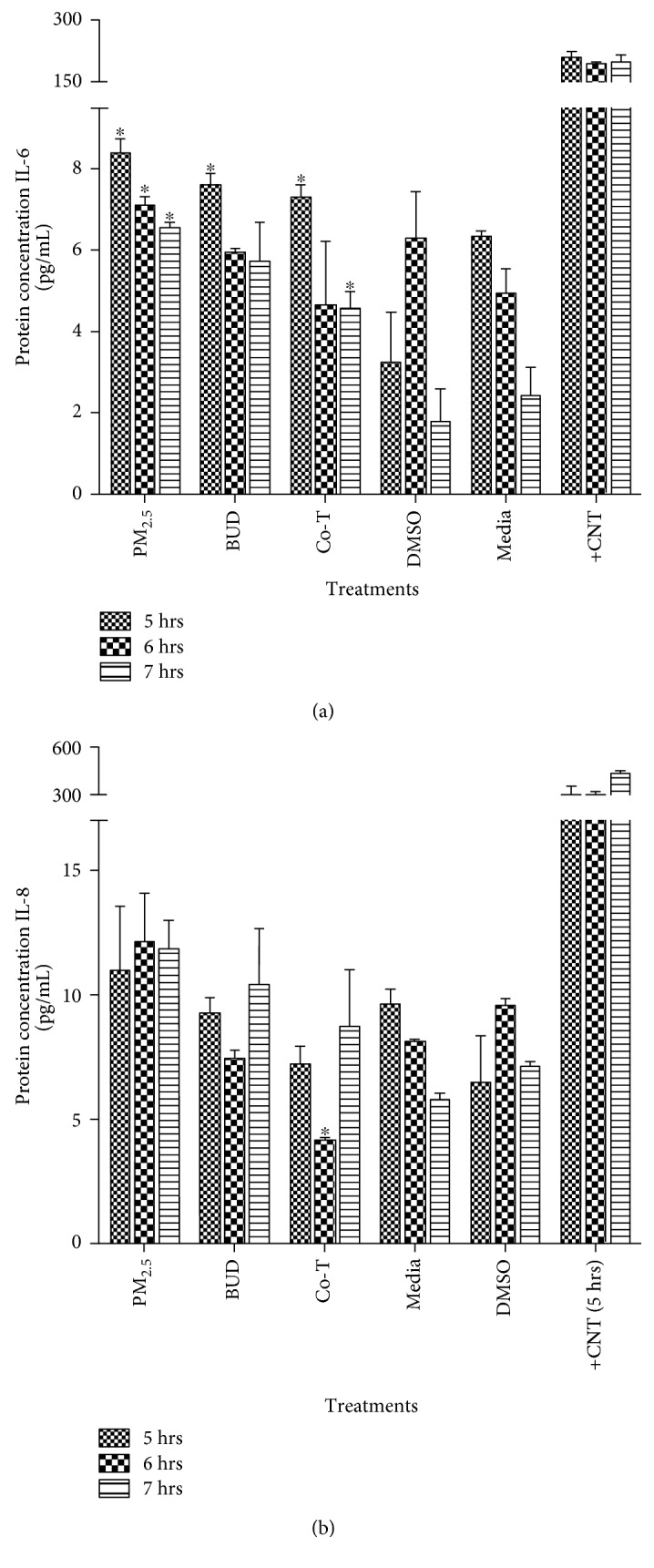
Induction of IL-6 and IL-8 in BEAS-2B exposed to PM_2.5_ extract, budesonide (BUD), and the Co-T. Cytokines were measured using a multiplex bead system and Luminex instrument, after 24 hr of exposure. Bars represent mean protein concentration ± SEM of three independent experiments (*N* = 3); ^∗^*p* < 0.05. Asterisk over the bar indicated the comparison of a treatment with DMSO. LPS (10 *μ*g/ml) was used as positive control.

After exposure to PM_2.5_ extract, IL-6 and IL-8 concentrations were assessed. Previous studies report induction of IL-6 and IL-8 at short periods of time after PM_2.5_ exposure [4]. Therefore, we selected the time points of 5, 6, and 7 hours as in the gene expression experiments. Our results show that IL-6 concentration decreases in a time-dependent manner after exposure to PM_2.5_ extract, BUD, and the Co-T (Figure 3(a)). The highest concentration of IL-6 was obtained at 5 hr and was the highest of any treatment. The cotreatment significantly reduced IL-6 secretion when compared to the effect of any of the individual treatments (Figure 3(a)). This inhibitory effect was also seen for IL-8 in cotreated cells at 6 hr when compared to PM_2.5_ extract and BUD treatments alone at the same exposure time. Concentrations of IL-13 and IL-10 with all the treatments described above after 24 hr in BEAS-2B were also evaluated; however, no significant changes in cytokine expression were observed. Slight increases in IL-8 at different time points were observed with the various treatments, but these were not significantly different from controls (Figure 3(b)). The most prominent finding in IL-8 was observed with the cotreatment at 6 hr where a significant reduction was reported.

## 4. Discussion

Exposure to PM_2.5_ causes exacerbation of several conditions of the respiratory system and cardiovascular diseases [5, 14, 40]. The results from the toxicity assays of PM_2.5_ extract showed nontoxic effects in BEAS-2B at 25 *μ*g/ml, as previously reported by Rodriguez-Cotto et al. and Akhtar et al. using lung cell lines [1, 5]. These studies also report that concentrations above 75 *μ*g/ml are toxic to lung cells, like our findings. A difference between this study and the one by Rodriguez-Cotto et al. was observed after their LD_50_ was taken into consideration [1, 5]. The toxicity of Guayama PM_2.5_ extract was significantly higher (LD_50_ = 76.7 *μ*g/ml) than that of Fajardo's (LD_50_ = 122 *μ*g/ml) [5]. The main reason for this is that Guayama is more likely an urban industrialized site while Fajardo is a rural site. Since the physicochemical properties of PM_2.5_ depend on its size and the source of origin, it was not surprising to find this trend between the different areas [1, 41]. In addition, the topographic and seasonal changes are different among sites, such as the African dust phenomenon that affect both sites in a different manner [5, 10, 42].

The BUD glucocorticoid treatment was employed to evaluate its effect on ABCC gene expression alone and in the Co-T. Within the recommendable doses of BUD reported in the literature is 0.1 *μ*g/ml to use in bronchial epithelial cells [43, 44]. However, we found this dose to be toxic for BEAS-2B; hence, the highest nontoxic concentration was determined to be 0.05 *μ*g/ml. The Co-T was nontoxic to BEAS-2B. Taking that into account, the possibility of a synergistic effect (between PM_2.5_ and BUD) enhancing cell proliferation or death was ruled out. The cells presented a normal proliferation after the Co-T exposure. This outcome allowed us to conduct the gene expression studies with the assurance that the cellular environment was reliable and not altered by apoptosis or related mechanisms.

PM_2.5_ exposure provokes an antioxidant rather than an inflammatory response. PM_2.5_ has been found to induce the release of immune mediators in BEAS-2B, as previously mentioned [4, 10, 45, 46]. It has been proven that this antioxidant response takes place because of the metals in the matrix of PM_2.5_ that provoke the induction of reactive oxygen species (ROS). Therefore, after a PM_2.5_ exposure, Nrf2 activates an upregulation of HMOX1 and GSTP1 genes that are essential to enhance the metabolic and antioxidant defense [4]. Nrf2 is responsible for detoxification and xenobiotic removal due to its role in activating the gene transcription of antioxidant and phase II detoxification enzymes, followed by phase III efflux transporters [45, 46]. It is important to highlight that the role of Nfr2 as a transcription factor inducing ABCC proteins has been studied in many fields [26, 47–49]. Accumulations of superoxides generate oxidative stress while the Nrf2 is activating in the cell cytoplasm. Nrf2 translocates into the cell nucleus thereby activating the antioxidant response elements (AREs), which encode the ABCC1–4 genes. Moreover, studies using small interfering RNA (siRNA) have shown a direct dependence among MRPs and Nrf2 during oxidative stress conditions [26, 50, 51]. Therefore, no doubt exists regarding the positive association between Nrf2 and MRPs in different scenarios where oxidative stress is the common variable.

These mechanisms have been observed in BEAS-2B, and our data support that the ABCC1 transporter may have an important role in PM_2.5_ metabolism. The cell antioxidant and protective responses include pumping out xenobiotics through the ABCC transporters, as previously mentioned. The ABCC transporters are recognized for their essential role in transporting glutathione s-conjugates, which is, thus, their importance on gene expression during oxidative stress [48, 52, 53]. Since all the xenobiotics were contained in the PM_2.5_, we hypothesized an increase in ABCC1, ABCC2, ABCC3, and ABCC4 gene regulation after PM_2.5_ exposure in BEAS-2B. Our results show a significant upregulation only for ABCC1 and ABCC4. This is the first report, to our knowledge, using BEAS-2B that demonstrated an ABCC1 upregulation due to the PM_2.5_ exposure.

ABCC1 downregulation and immune response suppression have been related with cigarette smoke extracts using lung cells and in animal studies [54, 55]. In addition, it has been reported that smokers with COPD have been found with a deficiency in MRP1 which is the resulting product from the ABCC1 translation [56, 57]. These findings are related with the cell response that we observed at 5 hr of PM_2.5_ exposure.

Studies in H69 lung cancer cells demonstrated that Nrf2 activated the MRP1 as a defense mechanism to promote cell survivor [58]; however, ABCC2 gene expression was not found in this cell line. The ABCC2 gene expression in lung cells has been debated [59]; here, we report nonsignificant induction in BEAS-2B. ABCC3 expression in lung cells has been also debated and reported differently within the same tissue including the lung tissue [60]. Nonsignificant results were found at any PM_2.5_ exposure time with ABCC3 gene. It is also known that ABCC3 is a close homologous of ABCC1 and found in some reports to be mutually excluded [61]. The ABCC3 transporter has a higher preference for glucuronide conjugates rather than for glutathione conjugates. Thus, the PM_2.5_ clearance depends on the antioxidant response and is logic to find ABCC1 upregulation instead of ABCC3. PM_2.5_ exposures also provoke an upregulation of ABCC4 at 5 hr. The ABCC4 protein is recognized as the versatile transporter within the ABCC family, because of its remarkable ability to transport a diversity of substrates. These ABCC4 substrates may include endogenous and xenobiotic organic anionic compounds, cyclic nucleotides, eicosanoids, urate, and conjugated steroids among others [62]. Moreover, our results suggest a possible role after the PM_2.5_ exposure in BEAS-2B. It is important to highlight that ABCC4 possesses a pathogenic role in the progression of pulmonary arterial hypertension (PAH) in humans [63]. More than one hypothesis was tested to screen for ABCC gene regulation by using the BUD and Co-T. The regulation of ABCC transporters with steroid drugs is more important since these drugs are commonly used to tackle inflammatory processes. The need to facilitate the transport of drug metabolites to and out of the cell is critical during treatment. Therefore, how these ABCC transporters are regulated in lung epithelial cells under BUD treatment is of great value. The BUD treatment upregulates the ABCC1 expression in BEAS-2B. In Calu-1 (lung cancer cells), this behavior is not noted [64]. Low concentration of BUD applied in cancer therapy inhibits the expression of vascular endothelial growth factor and MRP1 [65]. However, in the normal lung cell line 16HBE14o, BUD has been reported to upregulate ABCC1, supporting our research findings [66]. ABCC3 was not upregulated after BUD treatment. A significant downregulation of ABCC3 was detected at 6 hr, and then at 7 hr, its expression was stable. ABCC4 was also upregulated by BUD since MRP4 has been identified as a steroid transporter [59]. In asthma and COPD patient overexpresses, ABCC4 suggests that the steroid causes effects on its upregulation. We provide evidence that BUD generally increases ABCC1 and ABCC4 mRNA levels in epithelial lung cells during the first 6 and 7 hr of exposure. It is important to understand the dynamics of simultaneous exposure to particle pollution and inhaled corticosteroids since these are concurrently present during respiratory treatment. The effects of these two variables on ABCC regulation are an essential issue that needs to be addressed. ABCC1 was downregulated at 6 hr of Co-T exposure, opposing ABCC3 which was upregulated. The ABCC4 expression was upregulated by BUD treatment as well as PM_2.5_; thus, the cell recognizes the necessity and importance of its transport as a response to treatment.

Induction of cytokines by PM_2.5_ in lung cells has been previously reported [2–5, 10]. Airborne particulate matter contains a mixture of many organic and inorganic compounds, which induce a series of biochemical pathways and epigenetic changes that alter immune gene expression at different levels as a defense response to environmental insult [18, 37, 67, 68]. It has been reported that PM can induce IL-6 and IL-8 secretion in BEAS-2B by ROS and through the activation of NF-*κ*B or Nrf2 transcription factors [3, 4, 13, 48]. There is not much information in the literature to strongly support these findings by a cellular mechanism. Despite that, IL-6 and IL-8 have been detected after PM_2.5_ exposure while the NF-KB has been undetected [4]. IL-8 was not detected with PM_2.5_ at any of the time points. However, previous studies with PM_2.5_ organic extracts in BEAS-2B showed induction of IL-8 at 6 and 8 hr, supporting the release at a longer time. Contrary, the mRNA activation of IL-6 has been reported after 6 to 7 hr of PM_2.5_ exposure as well as what we reported in our time course [4]. Nrf2 directly regulates the mRNA of IL-8 in different types of cells [19]. IL-6 enhance the TH2 immune response mediated by lung epithelial cells and smooth muscle cells after the allergenic insult [69, 70]. The IL-6 overexpression was considered a byproduct of an ongoing inflammation, but recently has been recognized as a primary secreted cytokine in the epithelial cells [69]. IL-6 is also documented as one of the potential targets for the management and follow-up of chronic lung disease pathologies (e.g., asthma and COPD) [69, 71]. Since we found IL-6 in BEAS-2B, we can conclude that this response starts at an early stage of PM_2.5_ exposure in normal lung cells. Thus, our work supports that PM_2.5_ could lead to a major pathologic problem in the respiratory system.

Glucocorticoids are known potent regulators of inflammation and have been used pharmacologically against inflammatory, immune, and lymphoproliferative diseases for more than 50 years [34, 72]. However, glucocorticoids possess a broad variety and range of anti-inflammatory actions that are still not fully understood [72, 73]. We expected that PM_2.5_ will activate IL-6 and IL-8 in BEAS-2B as a proinflammatory response, and BUD was expected to decrease cytokine levels. Thus, we expected a decrease of the cytokines with the Co-T. Nonsignificant differences were detected in IL-8 between the cells treated with BUD and control. We found induction of IL-6 secretion in BEAS-2B by BUD rather than inhibition at 5 hr. IL-6 has pleiotropic function within different organs including the lungs [74–76]. Mechanistic studies demonstrated an IL-6 induction in airway smooth muscle cells after corticosteroid exposure [75]. This effect was only observed at 5 hr; after 6 to 7 hr, the IL-6 protein concentration decayed with no statistical significance. The Co-T had a significant suppression effect on IL-8, and this might be caused by the suppressive properties of the corticosteroids since PM_2.5_ did not stimulate IL-8 secretion by itself [77]. However, more experiments are needed to evaluate whether the Co-T has any influence in the IL-8 or IL-6 signaling pathways once the BEAS-2B are treated with PM_2.5_.

## 5. Conclusions

**Figure 4:**
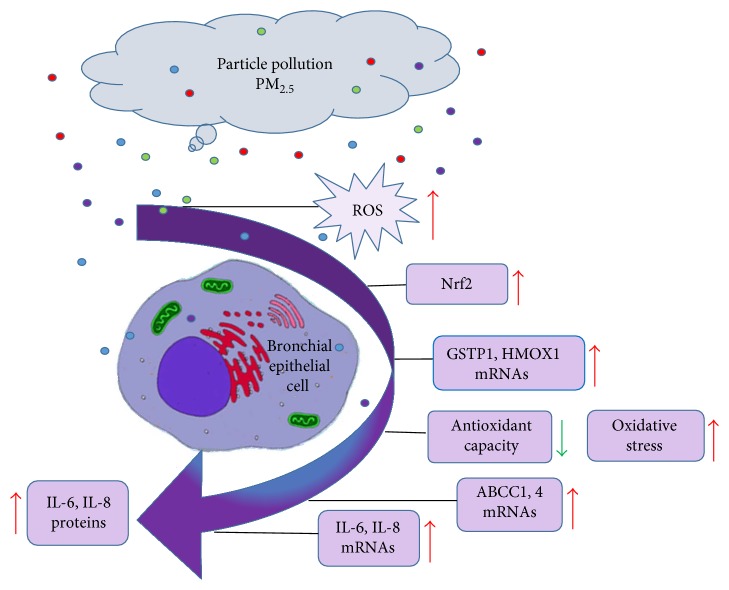
BEAS-2B respond to PM_2.5_ organic extracts. Local particle pollution has the capacity to generate ROS triggering the activation of Nrf2 and inducing the synthesis of antioxidant mRNAs: HMOX1 (heme oxygenase 1) and GSTP1 (glutathione-S-transferase). The antioxidant capacity is reduced provoking oxidative stress and the synthesis of ABCC1 and 4 and IL-6 and IL-8 mRNAs and their respective proteins.

PM_2.5_ activates the antioxidant mechanisms and the induction of ABCC1 and ABCC4 mRNAs in BEAS-2B (Figure 4). Since after the 24 hr of exposure with PM_2.5_ (25 *μ*g/ml), the cells were more than 80% viable, tempting to suggest that this finding is part of the management of xenobiotics metabolism in BEAS-2B. The Co-T exposure points out the need to perform more experiments to understand the signaling regulation in the lung cells to discriminate among ABCC1–4 gene transcription. However, most of the respiratory and cardiovascular diseases related to PM_2.5_ exposures or allergens have been associated with an ABCC and cytokine dysregulation [15, 55, 56, 59, 66]. This is the first report to our knowledge that studies the mRNA expression of ABCC1, 3, and 4 genes exposed to Co-T. Most of the work done with transporters has considered exposure to diesel particles and not to ambient PM. Future experiments must consider searching for posttranscriptional modifications to elucidate the mechanism that regulates Nrf2 in the ABCC gene transcription under PM_2.5_, BUD, and Co-T as well as study the activation of the MRP transporters. The cytokine experiments demonstrate an elevated expression of IL-6 at 7 hr with the PM_2.5_ that slightly decreases after the Co-T exposure. This fact could be indicating that the BUD could not perform its pharmacological task completely in the presence of PM_2.5_. Moreover, different mechanisms of action have been proposed and debating in the literature to explain the therapeutic and metabolic pathways associated with corticosteroids pharmacology [78, 79]. Although the inhibitory effects of corticosteroid therapy on the reproduction of osteoblast cells have been well elucidated [80], it is important to understand the fate of these corticosteroids after they are applied as therapy. Understanding the broad spectrum of molecule interaction between PM_2.5_ and the bronchial epithelial cell response will provide additional evidence to comprehend the PM_2.5_ role in the inflammatory process. It will also provide new avenues for innovative therapeutic approaches to benefit people over the world that are exposed to air pollutants.
